# Intracellular dynamics of archaeal FANCM homologue Hef in response to halted DNA replication

**DOI:** 10.1093/nar/gkt816

**Published:** 2013-09-17

**Authors:** Roxane Lestini, Sergey P. Laptenok, Joëlle Kühn, Mark A. Hink, Marie-Claire Schanne-Klein, Ursula Liebl, Hannu Myllykallio

**Affiliations:** ^1^Laboratoire d’Optique et Biosciences, Ecole Polytechnique, CNRS UMR7645–INSERM U696, 91128 Palaiseau Cedex, France and ^2^Section of Molecular Cytology, Swammerdam Institute for Life Sciences, van Leeuwenhoek Centre for Advanced Microscopy, University of Amsterdam, Science Park 904, 1098 XH Amsterdam, The Netherlands

## Abstract

Hef is an archaeal member of the DNA repair endonuclease XPF (XPF)/Crossover junction endonuclease MUS81 (MUS81)/Fanconi anemia, complementation group M (FANCM) protein family that in eukaryotes participates in the restart of stalled DNA replication forks. To investigate the physiological roles of Hef in maintaining genome stability in living archaeal cells, we studied the localization of Hef–green fluorescent protein fusions by fluorescence microscopy. Our studies revealed that *Haloferax volcanii* Hef proteins formed specific localization foci under regular growth conditions, the number of which specifically increased in response to replication arrest. Purification of the full-length Hef protein from its native host revealed that it forms a stable homodimer in solution, with a peculiar elongated configuration. Altogether our data indicate that the shape of Hef, significant physicochemical constraints and/or interactions with DNA limit the apparent cytosolic diffusion of halophilic DNA replication/repair complexes, and demonstrate that Hef proteins are dynamically recruited to archaeal eukaryotic-like chromatin to counteract DNA replication stress. We suggest that the evolutionary conserved function of Hef/FANCM proteins is to enhance replication fork stability by directly interacting with collapsed replication forks.

## INTRODUCTION

The maintenance of genome integrity is a crucial challenge for all proliferating cells. Consequently, elaborated DNA repair pathways to counteract and repair DNA damage caused by toxic by-products of cellular metabolism and/or environmental factors have evolved during the evolution of free living organisms. Unrepaired DNA lesions may impede progression of DNA replication complexes (replisomes), thus preventing faithful duplication of genetic material. Mechanistic details for the restart of stalled forks and how this results in genome rearrangements have been described in bacteria (1), yeast (2) and higher eukaryotes (3). The picture that emerges from numerous studies is that bacterial and eukaryal proteins implicated in homologous recombination also play a key role in stabilizing and/or restoring blocked replication forks. In agreement with this notion, inhibiting the elongation phase of DNA replication increases the frequency of replication-coupled recombination as a result of the accumulation of four-branched DNA intermediates that occur during reversal of the blocked replication forks (4).

To date little is known regarding the repair of stalled replication forks in archaeal species. Archaea, representing the third domain of life, are frequently found in extreme environments and must therefore be able to replicate and maintain their genomes intact under deleterious conditions such as elevated temperature, high salt concentrations, pH shifts and ionizing radiation. As archaeal replication proteins are evolutionarily related to their eukaryotic counterparts (5–7), studies on how archaea handle blocked replication forks have potential to increase our evolutionary understanding of the rescue of arrested replication forks. It was recently shown that two pathways may exist for restarting stalled replication forks in *Haloferax volcanii*, a salt-loving euryarchaeon (8). In particular, this study demonstrated that the *H. volcanii* Hef helicase/endonuclease from the DNA repair endonuclease XPF (XPF)/Crossover junction endonuclease MUS81 (MUS81)/Fanconi anemia, complementation group M (FANCM) family of endonucleases (helicase-associated endonuclease fork-structure DNA) is essential for cell viability only in the absence of the Holliday Junction resolvase Hjc (8). As single *hef* or *hjc* deletion mutants did not present an obvious growth or recombination phenotype, the synthetic lethality of the double *hef hjc* deletion mutant suggested that these two genes define two parallel pathways for cell viability and DNA repair in *H. volcanii*.

XPF/MUS81/FANCM proteins are structure-specific endonucleases that act on D-loop, splayed-arm and replication fork DNA substrates during DNA replication, repair and recombination. Among this large, intensively studied, nuclease family, archaeal Hef proteins are unique as they contain active helicase and nuclease domains that *in vitro* are required for the rearrangement of fork-structured DNA (9) and *in vivo* are necessary for cell viability in the absence of Hjc Holliday function resolvase (8). In *Pyrococcus abyssi*, a hyperthermophilic archaeon, a protein complex including Hef (PAB1090), the replication clamp, the replication factor C (the clamp loader) and endonuclease NucS has been identified (10,11). The structures of the helicase and the nuclease domains of *Pyrococcus furiosus* Hef have been resolved using radiographic crystallography, but to date no biochemical or structural information exists on the Hef holoprotein. It is of note that Hef proteins have not been found in bacteria, but are considered orthologous to the human Fanconi anemia protein FANCM (12,13). Hence, investigating Hef proteins *in vitro* and/or *in vivo* has potential furthering our understanding of the molecular functions of their eukaryotic homologs that are associated with human diseases such as Fanconi anemia and *Xeroderma pigmentosum* (14).

To get direct insight into the role of Hef in maintaining genome stability in *H*. *volcanii* living cells, we studied the localization of Hef–green fluorescent protein (GFP) fusions by fluorescence microscopy. Our studies revealed that Hef proteins formed specific localization foci under regular growth conditions, and that the number of these foci specifically increased in response to replication arrest. Using life cell imaging, we also investigated the dynamic behavior of these foci in archaeal cells. Purification of the full-length Hef protein from its native host revealed that it forms a stable homodimer in solution, with a peculiar elongated configuration. Altogether, our data indicate that the shape of Hef, significant physicochemical constraints and interactions with DNA limit the apparent cytosolic diffusion of halophilic DNA replication/repair complexes, and demonstrate that Hef proteins are dynamically recruited to archaeal eukaryotic-like chromatin to counteract DNA replication stress. Our studies indicate that Hef/FANCM proteins may enhance replication fork stability by directly interacting with collapsed replication forks.

## MATERIALS AND METHODS

### Chemicals

Unless stated otherwise, all chemicals used were from Sigma Biolabs.

### Molecular biology techniques

Isolation of genomic and plasmid DNA and transformation of *H. volcanii* were carried out using published protocols (8,15,16). Standard molecular biology techniques were used for DNA isolation and manipulation. All enzymes were purchased from New England Biolabs.

### Strains, plasmids and growth conditions

*Escherichia coli* strains XL1-Blue MRF’ [ΔmcrA183 ΔmcrCB-hsdSMR-mrr173 endA1 supE44 thi-1 recA1 gyrA96 relA1 lac (F′ proAB lacI^q^ZΔM15 Tn10)] and GM121 (F^-^dam-3 dcm-6 ara-14 fhuA31 galK2 galT22 hdsR3 lacY1 leu-6 thi-1 thr-1 tsx-78) were used for cloning. The latter strain was used to prepare unmethylated plasmid DNA for efficient transformation of *H. volcanii*.

*Haloferax volcanii* strains used and constructed during this work are described in Table 1. *Haloferax volcanii* cultures using enriched Hv-YPC or Hv-Ca media were grown at 45°C, as described previously (15). Different drugs were added to overnight cultures that were diluted to OD_600_ ≈0.1 after 2 h growth at 45°C, and incubation was continued for 18 h. Where indicated, different compounds were added to liquid cell cultures at the following concentrations: aphidicolin (1, 2.5, 5 or 10 µg/ml dissolved in 100% Dimethyl Sulfoxide (DMSO)), mitomycin C (MMC; 0.075 µg/ml), phleomycin (0.1 µg/ml) and hydroxyurea (HU; 5 mM). When aphidicolin stock solution was added to cells, negative controls using DMSO only were analyzed in parallel. For MMC plate assays, overnight cultures were streaked on solid Hv-YPC media in the presence and absence of 0.02 µg/ml MMC, followed by 7 days incubation at 45°C. To determine the fraction of surviving cells, cultures were diluted in 18% salt water and 20 µl aliquots were spotted on Hv-YPC plates. Individual colonies were counted after 4 days, except for plates of the slow growing strain HvRL61 (Δ*pyrE2 hef^+^::gfp^+^* Δ*hjc*) that were incubated 7 days before counting.

**Table 1.** *Haloferax volcanii* strains used

Strain	Relevant genotype	Source or reference
H26	Δ*pyrE2*	(15)
H178	Δ*hjc*	(8)
H358	Δ*hef*	(8)
H1209	Δ*hdrB* Δ*mrr pitA_Nph_*	(15)
HvRL8	Δ*hdrB* Δ*mrr pitA_Nph_*Δ*hef*	(pTA370)
HvRL26	Δ*hdrB* Δ*mrrpitA_Nph_* Δ*hef {p.tnaA::6xHis tag::hef^+^ pyrE2^+^ hdrB^+^}*	HvRL8 × pRL6
HvRL37	Δ*pyrE2hef*^+^::*gfp*^+^	(pRL12)
HvRL61	Δ*hjc*	(pTA225)
HvRL65	Δ*hef gfp*^+^ inserted at *hef* locus	(pRL32)
HvRL66	Δ*hef gfp^+^::gfp*^+^ inserted at *hef* locus	(pRL34)

### Construction of mutant strains and expression plasmids

Deletion mutants were constructed using the pop-in/pop-out method as described previously (8,16). Table 2 lists plasmids used for gene deletion and protein expression studies that were polymerase chain reaction (PCR) amplified using different primer combinations (Table 3). The template DNA used for PCR amplification was either isolated plasmid DNA or an isolated *H. volcanii* colony resuspended in 100 µl of sterile water.

**Table 2.** Plasmids used

Plasmid	Relevant properties	Source or reference
pTA131	Integrative vector based on pBluescript II, with *pyrE2* marker	(15)
pTA225	pGB70with Δ*hjc* construct, generated by deletion of 463 bp *Psh*AI fragment of *hjc* gene from 2325 bp *Xma*I-*Xmn*I subclone of chromosomal fragment in pTA48	(8)
pTA370	pTA131 with 1.6 kb Δ*hef* construct, consisting of upstream *Kpn*I-*Bam*HI PCR fragment and downstream *Bam*HI-*Xba*I PCR fragment ligated and inserted at *Kpn*I-*Xba*I sites	(8)
pTA963	Overexpression vector with *p.tnaA*::6xHis tag, *pyrE2* and *hdrB* makers, and pHV2 origin	(17)
pTA1097	pTA131 with 4.7 kb *Apa*I-*Not*I fragment of pTA334 containing *hef* gene and flanking regions	(8)
pJAM1020	Ap^r^ Nov^r^, smRSGFP expressed in *H. volcanii*	(18)
pRL6	pTA963 with a PciI-EcoRI *hef*^+^ PCR product inserted	This study
pRL12	pTA1097 with insertion of a *gfp*^+^ PCR fragment at BlpI site after restriction and Mung nuclease treatment to obtain blunt extremities	This study
pRL29	pBluescript vector with a synthetic *gfp* gene encoding smRSGFP with a codon bias optimized for *H. volcanii*(Genecust)	This study
pRL32	Integrative vector pTA131 with insertion of flanking regions of *hef* gene with the *gfp* gene under the control of *hef* promoter	This study
pRL34	Integrative vector pTA131 with insertion of flanking regions of *hef* gene with two *gfp* genes in tandem under the control of *hef* promoter	This study

**Table 3.** Oligonucleotides used

Primer	Sequence (5′–3′)^a^	Relevant properties	Plasmid
RL31	GGCAACCGCGAGGACTGAG	Amplification of *hjc* chromosomal locus	
RL32	CGAGATGGTCGGCGGGATG	Amplification of *hjc* chromosomal locus	
RL33	GAGACGAACGCCGACTAC	Amplification of *hef* chromosomal locus	
RL34	GTGGGAGACGCTCAGAAC	Amplification of *hef* chromosomal locus	
RL39	TCACACATGTCGGCCTCCGAGGACG	*hef ^+^*amplification from pTA1097*, PciI* site	pRL6
Hef 3′ R	CGACGAATTCGTGATGGGCCACC	*hef ^+^*amplification from pTA1097*, EcoRI* site	pRL6
RL54	ATGAGTAAAGGAGAAGAAC	*gfp^+^*amplification from pJAM1020	pRL12
RL55	TTATTTGTATAGTTCATCC	*gfp^+^*amplification from pJAM1020	pRL12
RL85	ATGAGTAAAGGAGAAGAACTTTTC	*gfp^+^*amplification from pJAM1020	pRL32/34
RL85bis	ATGTCGAAAGGCGAGGAACTCTTC	*gfp^+^*amplification from pRL29	pRL34
RL108	**ATCGATAAGCTTGAT**CGGCAACCGCGAGGACTG	*hef* US region amplification from pTA1097	pRL32/34
RL109	**CTCGCCTTTCGACAT**CGGTGACGATTGCTCG	*hef* US region amplification from pTA1097	pRL34
RL114	GCCGCGTTCACCGCCCGGAGC	*hef* DS region amplification from pTA1097	pRL32/34
RL115	**CTGCAGGAATTCGAT**CGAACCGGAGCTTTCGAC	*hef* DS region amplification from pTA1097	pRL32/34
RL116	**GGCGGTGAACGCGGC**TTATTTGTATAGTTCATC	*gfp^+^*amplification from pJAM1020	pRL32/34
RL117	**TTCTCCTTTACTCAT**CGACCGGTAGGCGTAGC	*gfp^+^*amplification from pRL29	pRL34
RL119	**TTCTCCTTTACTCAT**CGGTGACGATTGCTCG	*hef* US region amplification from pTA1097	pRL32

^a^Restriction endonuclease sites used in cloning are underlined, 15 bases overlap with flanking fragments are shown in bold.

**Figure 1.**
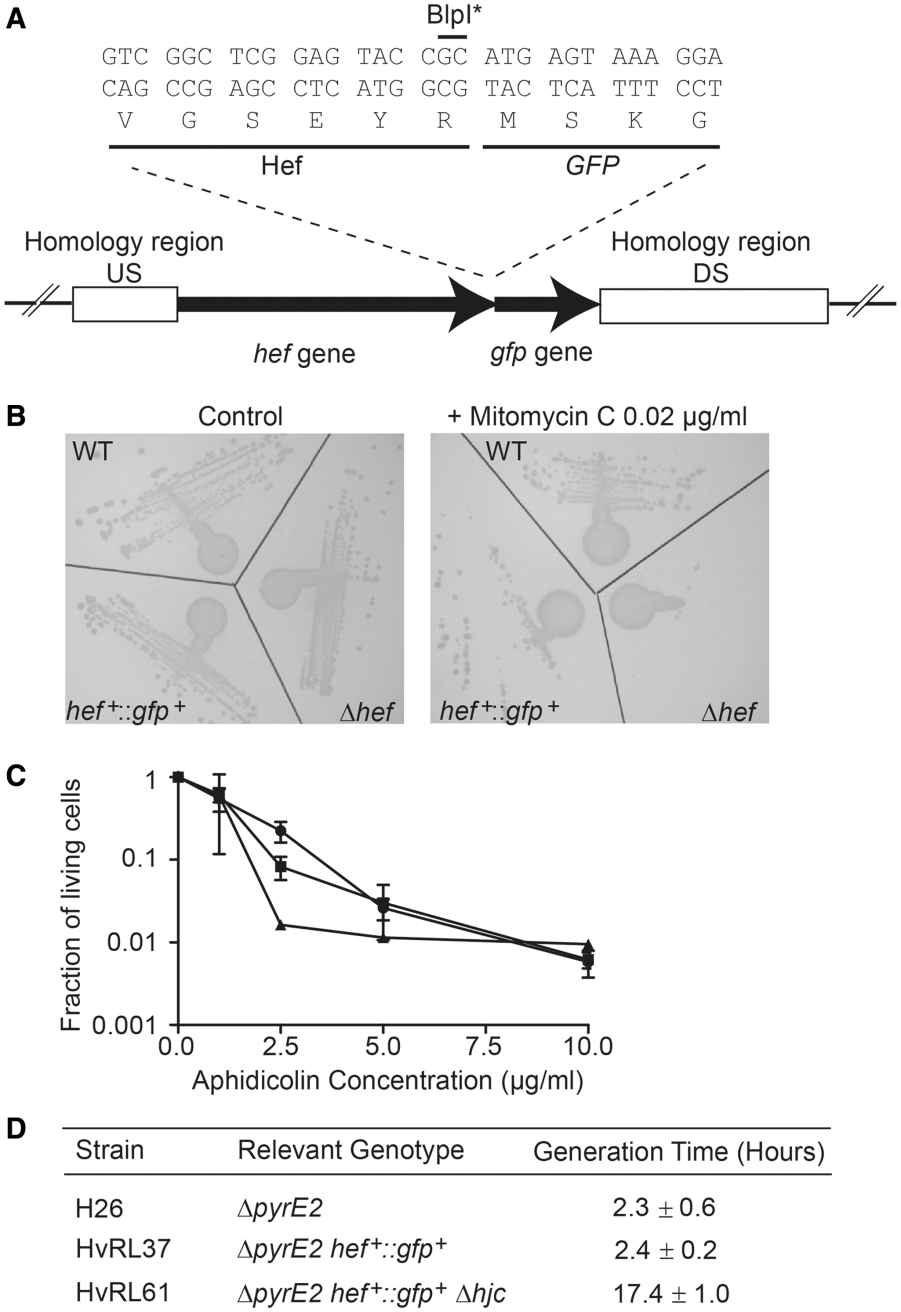
*gfp*-fused *hef* allele construction and functional characterization. (**A**) Representation of the chromosomal locus of the *hef*::*gfp* allele. The regions of homology between the plasmid and the chromosome used for pop-in/pop-out gene replacement are represented by white boxes (Upstream region and downstream region). The sequence of the 3′ end of the *H.volcanii hef* gene fused to the *gfp* gene is shown. BlpI* refers to the inactivated restriction site after cleavage and processing of the cohesive extremity into blunt end. (**B**) Strains streaked on YPC control plates and YPC plates containing 0.02 µg/ml MMC after 7 days of incubation. (**C**) Fraction of living cells in response to increasing concentrations of aphidicolin for WT (black square), *hef*-deleted (black triangle) and *hef*^+^::*gfp*^+^ cells (black circle). Error bars represent standard deviations of at least three independent experiments. (**D**) Generation times (± SEM) of WT and *hef*^+^::*gfp*^+^ cells, and *hef*^+^::*gfp*^+^ in a *hjc*-deleted background.

The fusion plasmid pRL12, encoding the carboxy-terminally GFP-tagged Hef protein, was constructed using the plasmid pTA1097 carrying *hef* and its promoter sequence (8) and exploiting the presence of a unique *Blp*I site overlapping with the STOP codon of *hef*. First, linearized pTA1097 plasmid with 5′ overhangs was created using a *Blp*I restriction enzyme. The obtained plasmid was then treated with Mung bean nuclease to obtain a DNA fragment with blunt ends. Finally, the blunt-ended GFP fragment obtained by PCR was ligated to the blunt-end vector, thus creating an in-frame *hef*::GFP fusion (Figure 1A). The smRS-GFP fragment was obtained by PCR on pJAM1020 plasmid (18) using RL54 and RL55 oligonucleotides. The DNA sequence of the resulting fusion-plasmid was confirmed by DNA sequencing on both strands.

Two plasmids were constructed to allow the specific expression of monomeric or dimeric GFP under the control of the *hef* promoter at the *hef* chromosomal locus. pRL29 is a pBluescript derivative with the insertion at *Not*I and *EcoR*I sites of a synthetic gene encoding smRSGFP followed by a linker sequence (GEGQGQGQGPGRGYAYRS) with a codon bias optimized for expression in *H. volcanii*. The two *gfp* alleles from pJAM1020 and pRL29 are not identical, explaining why they are simultaneously stable in the *H*. *volcanii* genome. The plasmid pRL32 was generated using the In-Fusion HD Cloning kit (Clontech) by inserting into the *Eco*RV-linearized pTA131 plasmid three PCR-generated fragments with 15-bp overlaps at their ends: (i) the *hef* upstream region amplified from pTA1097 using RL108 and RL119, (ii) the *gfp* gene amplified from pJAM1020 using RL85 and RL116 to allow expression of *gfp* under the control of the *hef* promoter and (iii) the *hef* downstream region amplified from pTA1097 using RL114 and RL115. The plasmid pRL34 was generated using the In-Fusion HD Cloning kit (Clontech) by inserting into the EcoRV-linearized pTA131 plasmid four PCR-generated fragments with 15-bp overlaps at their ends: (i) the *hef* upstream region amplified from pTA1097 using RL108 and RL109, (ii) the *gfp* gene amplified from pRL29 using RL85bis and RL117, (iii) the *gfp* gene amplified from pJAM1020 using RL85 and RL116 to allow expression of tandem *gfp* under the control of the *hef* promoter and (iv) the *hef* down-tream region amplified from pTA1097 using RL114 and RL115. The sequence of the resulting plasmids was confirmed by DNA sequencing on both strands.

The expression vector pTA963 was used to express the *H. volcanii* Hef protein under the control of the tryptophan-inducible *tnaA* gene promoter (17). *Haloferax volcanii hef* was amplified from the plasmid pTA1097 (8) using RL39 and Hef 3′R oligonucleotides. The resulting PCR fragment was cloned into pTA963 using *Pci*I and *EcoR*I restriction sites included in the oligonucleotides. The resulting construct encodes the full-length protein carrying a six-histidine tag at its N-terminus. To prevent recombination between the chromosome and the expression plasmid, the expression construct was transformed into *H. volcanii* strain H1209 lacking *hef*. Protein expression was induced by adding tryptophan to exponential cell cultures to a final concentration of 5 mM. After 5 h the cells were collected by centrifugation and processed as indicated below.

### Wide-field fluorescence microscopy

Cells were mounted on glass slides covered with a thin layer of 1% agarose prepared in 18% salt water. Differential interference contrast (DIC; Nomarski interference contrast) and fluorescence images were obtained at room temperature using a ZEISS Axio Observer equipped with a 40×, 1.3 NA oil immersion objective. 470 nm excitation at maximum available intensity (4 W cm^−^^2^) and a filter set 65 HE (EX BP 475/30, BS FT 495, EM BP550/100) were used for fluorescence imaging. A maximum intensity z-projection of six slices (out of 30 slices centered on DIC focus—Δz = 0.250 µm) was performed using public domain open source software ImageJ (http://rsbweb.nih.gov). Quantitative image analyses were performed for each fluorescence image using IMARIS software 7.4.2. The following IMARIS software parameters were used during automated image processing to detect individual cells: 1.5 µm smooth filter width, 1 µm background subtraction sphere diameter, activated split cells by seed points and 2 µm cell seeds estimated diameter. When cells treated with 5 and 10 µg/ml aphidicolin were analyzed, a 4 µm cell seeds estimated diameter was used, reflecting the increased cell size during these conditions. Fluorescence foci within detected cells were automatically detected as ‘vesicles’ using the following parameters: 0.75 µm estimated diameter, no background subtraction, and a ‘quality’ filter without automatic threshold. For each detected cell, cell surface area, total fluorescence intensity, the number of fluorescence foci and their total fluorescence intensity were recorded. For all experiments, large number of images were analyzed by IMARIS and added for further analysis, and for each condition tested at least three independent experiments were analyzed. For each independent experiment the average cell surface (total cell surface/total number of cells), the mean intensity per cell (total cell fluorescence intensity/total number of cells), the mean intensity per cell surface (total cell fluorescence intensity/total cells surface), the average number of fluorescence foci per cell (total number of vesicles/total number of cells), the average intensity of fluorescence foci (total vesicles intensity/total number of vesicles), the proportion of fluorescence intensity at fluorescence foci (total vesicles intensity/total cell fluorescence intensity) and the relative frequency of cells with 0–20 fluorescence foci were determined. Student *t*-tests were performed using GraphPad Prism 5 software.

### Number and brightness measurements

To determine the brightness of Hef::GFP foci, we used number and brightness (N&B) analysis, a fluctuation-based fluorescence microscopy method (19). N&B experiments and statistical analyses of fluorescence fluctuation amplitudes were performed as described previously (20). Overnight cultures, started from a single isolated colony, were diluted to OD_600_ ≈ 0.1. Cells were incubated 2 h, then 5 µg/ml aphidicolin or the corresponding amount of DMSO as control were added and cultures were incubated for 18 h. To immobilize cells, 100 µl of cell culture were spotted on a poly-d-lysine–coated glass cover slip that was subsequently incubated for 20 min at room temperature. Images were acquired on an Olympus IX81 microscope equipped with a FluorView FV1000 scan and confocal detection head coupled to a custom-made Picoquant detection unit containing Micro photo devices (MPD) avalanche photodiodes working in single photon counting mode. The 488 nm excitation light from a CW Argon laser (Melles-Griot) was focused into the sample with a 60× UPLS Apochromat, 1.2 NA water immersion objective. Fluorescence passed through a 525DF45 bandpass filter (Chroma) placed just before the detector. The laser intensity was typically set at 9 kW cm^−^^2^. For a typical measurement, images of 128 × 128 pixels, with a pixel dimension of 207 nm, were acquired. The pixel dwell time was set at 100 μs. Image time-stacks of 100 image scans were collected at time intervals of 1.95 s per frame. Using an in-house developed program that was written in C++ (Qt 4.7.0), the Picoquant.pt3 data files containing photon arrival times were converted to intensity image-time Tiff stacks. These image stacks were analyzed using a custom written N&B ImageJ macro in which the equations from Digman *et al.* (19) were implemented. The apparent number and brightness for each pixel at position (*x*, *y*) were calculated (20). Cells that displayed obvious movement, resulting in experimental artifacts, were excluded from the analyses. Average molecular N&B values and corresponding standard errors of the mean were obtained from manually selected regions of interest (ROIs). Multiple ROIs, each containing a single fluorescence focus, were analyzed. As additional control, cells excluding fluorescence foci were analyzed in parallel.

### Fluorescence recovery after photobleaching measurements

The Olympus tornado-scanning feature was used for fluorescence recovery after photobleaching (FRAP) experiments. The samples were prepared and measured on an Olympus FV1000 confocal microscope as described for N&B analyses. 64 × 64 pixels images of cells were acquired with 276 nm pixel size and 40 µs dwell time. A circular region at one tip of the rod-shaped archaeal cell [21 ± 5% (3.72 ± 1.95 µm^2^of the cell)] was selected and bleached with a 488 nm CW Argon laser at 100% laser-intensity (200 kW cm^−^^2^) in tornado-scanning mode for a total bleach duration of 0.1 s. Bleaching was performed from the center outward and, in total, 150 images were collected including 50 prebleach frames at 0.272 s/frame. Background intensity trace, *I_b_(t)* was obtained from an ROI outside of the cell. The raw fluorescence intensity of the bleached area *I(t)* was normalized using 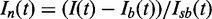, with *I_sb_(t)* serving as reference trace and was constructed from an exponential fit of fluorescence intensity of 50 pre-bleach frames corrected for the background. This normalization procedure results in traces where the pre-bleach intensity equals unity. The average of normalized traces from different experiments (17 in case of non-Aphidicolin (APD) and 26 in case of APD) was then fitted to the sum of exponential functions for a semiquantitative analysis as described in (21). A mono-exponential function was used to fit the recovery measured in control cells, whereas a bi-exponential function was required for aphidicolin-treated cells. Recovery constant k (s^−^^1^) was directly obtained from the fit and converted into the recovery half-time τ_1/2_ (s) calculated as τ_1/2_ = ln(2)/k. The 2D diffusion constant D (µm^2 ^s^−^^1^) was approximated according to D = (β × A)/(4 × τ_1/2_) with β = 1 for confocal microscope and the bleached area A estimated at 3.72 µm^2^.

### Affinity purification of *H. volcanii* Hef protein

*Haloferax volcanii* cells were lysed in buffer A (10 mM Hepes, pH 7.0, 2M KCl, 20 mM imidazole) by sonication on ice. Cellular debris and unlysed cells were eliminated by centrifugation (60 min at 18 000 g, 4°C). Tagged proteins were absorbed on Ni-NTA agarose (Qiagen) in a batch mode. Several washing steps using buffer A were performed and proteins bound to Ni-NTA agarose were eluted using buffer B (10 mM Hepes, pH 7.0, 2M KCl, 500 mM Imidazole). An ÄKTA10 purifier 10 system was used for further purification and removal of imidazole on an S-200 gel-filtration column (GE Healthcare Life Science) that was equilibrated using buffer C (10 mM Hepes, pH 7.0, 2M KCl). The purified protein was concentrated using a vertical membrane Amicon Ultra-15 centrifugal filter unit (Merck Millipore), with a 10 kDa cutoff. Typically, purified Hef proteins had a concentration of 3 mg/ml. Samples were stored at 4°C in buffer C.

### Analytical ultracentrifugation

Before analytical ultracentrifugation (AUC) analyses, large protein aggregates were removed by centrifugation at 16 000*g* for 30 min. Sedimentation velocity experiments were performed at 20°C in two-channel 12-mm cells, using a Beckman XLA70 ultracentrifuge operated at 32 000 rpm (65 450*g*) with an An-60Ti rotor. Sedimentation was followed by measuring protein absorption at 280 nm. AUC was performed on 0.9 mg/ml, 1 mg/ml and 1.6 mg/ml samples (9.6, 10.6 and 17.0 µM, respectively) in buffer C (2M KCl, 10 mM Hepes, pH 7.0). A viscosity of 1.0011 cp and a density of 1.08915 g/ml were used for buffer C calculated by SEDNTERP software (http://www.jphilo.mailway.om/). The sedimentation coefficients and the frictional ratio ƒ/ƒ^0^ were determined using SEDFIT software (www.analyticalultracentrifugation.com) (22).

Sedimentation equilibrium experiments used 0.6 or 1 mg/ml (10.6 or 6.4 µM, respectively) of *Hvo*Hef protein in buffer C. For sedimentation equilibrium experiments, a Beckman XLA70 ultracentrifuge was operated successively at 7000 rpm (3564*g*), 8400 rpm (5132*g*) and 12 100 rpm (10 649*g*) with a An-60Ti rotor, using absorbance optics at 280 nm. Data obtained were analyzed using SEDPHAT software (22).

### Western immunoblotting

Before cell lysis, cultured cells were diluted in 18% salt water and 20 µl aliquots were spotted on Hv-YPC plates, thus allowing the precise determination of the number of cells that were analyzed by western immunoblotting. Individual colonies were counted after 4 days. *Haloferax volcanii* cells were lysed in 2M KCl, 10 mM Hepes, pH 7.0, by sonication on ice. Cellular debris and unlysed cells were eliminated by centrifugation (60 min at 18 000*g*, 4°C). Cell lysates were separated on 4–12% sodium dodecyl sulphate-polyacrylamide gel electrophoresis (SDS-PAGE; Invitrogen) and transferred onto nitrocellulose membranes. Membranes were blocked with 50% Licor Blocking solution (Sciencetec) in phosphate buffered saline containing 0.1% Tween 20 and probed with anti-GFP antibodies raised in rats (1:4000, Chromotek). Antigen–antibody binding was detected with anti-rat IgG labeled with IRDye680 (1:5000, Sciencetec). Membranes were analyzed and quantified using a Licor Odyssey Imaging system (Supplementary Figure S1). To precisely quantify the amount of GFP, a calibration curve was established using commercial purified rGFPuv (Clontech).

## RESULTS

### Functional expression of GFP-tagged Hef protein in *H*. *volcanii* cells

To investigate the intracellular localization dynamics of *H. volcanii* Hef proteins, we first constructed the plasmid pRL12 (Table 2) that carries the *hef*^+^*::gfp*^+^ fusion protein together with its flanking chromosomal regions. The resulting construct was integrated in the chromosome of *H. volcanii* strain H358 (Δ*pyrE2* Δ*hef*) using the pop-in/pop-out method (Figure 1A). All colonies resulting from excision of the plasmid from the chromosome (pop-out) showed comparable growth characteristics on solid media. In 60% of the clones tested the *hef^+^*::*gfp^+^* fusion was inserted at the chromosomal *hef* locus, thus allowing the expression of a GFP-tagged Hef protein from its native promoter. One representative clone, dubbed HvRL37 (*hef*^+^*::gfp*^+^), grew similarly to wild type on solid or liquid media. We note that the smRS-GFP protein used for this Hef–GFP fusion is the only GFP variant that has been shown to be functional in *H. volcanii* cells (17). Following the same procedure, two control strains were constructed: HvRL65, expressing only the *gfp* gene from the *hef* promoter at the chromosomal locus (pop-in/pop-out of pRL32 in H358), and HvRL66, expressing a dimeric *gfp* variant encoded by a single gene from the *hef* promoter. Both strains showed wild type–like growth.

The *H. volcanii* Δ*hef* strain grows slowly on solid medium in the presence of the DNA damaging agent MMC (8). To investigate whether the GFP-tagged Hef protein is able to rescue this growth defect, we subjected the HvRL37 (*hef*^+^*::gfp*^+^) strain together with a wild type control strain (H26) to 0.02 µg/ml MMC in solid medium. Expectedly the Δ*hef* strain did not thrive under these conditions, while wild type and *hef*^+^::*gfp*^+^strains formed isolated colonies in the presence of MMC (Figure 1B), indicating rescue of the growth defect. To further test the functionality of the Hef::GFP fusion protein, we grew wild type, Δ*hef* and *hef*^+^*::gfp*^+^ strains in the presence of different concentrations of aphidicolin, a tetracyclic diterpene antibiotic that inhibits DNA synthesis in halophilic archaea (23). Cells were exposed to aphidicolin during the exponential growth phase for seven to eight generations (≈18 h) and plated on rich media and cell viability was compared with control cells treated with DMSO only (Figure 1C). At a maximal aphidicolin concentration of 10 µg/ml we observed a significant and dose-dependent decrease in cell viability for all tested strains (Figure 1C). We found the Δ*hef* strain to be substantially more sensitive to aphidicolin than wild type and *hef*^+^*::gfp*^+^ strains. This effect was most obvious at an aphidicolin concentration of 2.5 µg/ml, where the Δ*hef* strain was one order of magnitude more sensitive to aphidicolin than the two other strains tested. Because *hef*^+^::*gfp*^+^ and wild type cells behaved similarly under these experimental conditions, our results revealed that both, *Hvo*Hef and Hef::GFP proteins, reestablish DNA replication and cell viability after aphidicolin treatment (Figure 1C).

Although these results indicate that the Hef::GFP fusion protein is functional in repair of DNA damages caused by MMC and aphidicolin, we do not exclude the possibility that Hef proteins might have additional roles in DNA replication or other cellular processes in *H. volcanii*. To investigate this possibility further and prompted by an earlier demonstration that the Holliday junction resolvase HjC is essential for cell viability in the absence of Hef (8), we attempted to delete *hjc* in *hef*^+^::*gfp*^+^cells. In this experiment, two different colony types were observed on ‘pop-out’ plates: ‘normal’ and ‘small’, which corresponded to 92 ± 2% and 9 ± 3% of the total population, respectively. We tested four out of 17 ‘small’ colonies (24%) and found them to be Δ*hjc*, whereas all ‘normal’-sized colonies were *hjc*^+^. The growth of two ‘small’ Δ*hjc hef*^+^::*gfp*^+^ colonies was further studied in liquid and on solid rich media, revealing no differences. Work was continued using a representative Δ*hjc hef*^+^::*gfp*^+^ clone named HvRL61 that expresses GFP-tagged Hef from the native chromosomal locus in a Δ*hjc* background. As the ‘small’ size of the *hef*^+^::*gfp*^+^Δ*hjc* colonies suggested a growth defect of the strain, we compared its generation time with wild type and *hef*^+^::*gfp*^+^ strains. We found a generation time of *hef*^+^::*gfp*^+^Δ*hjc* in rich medium that was 5–6 times longer than what was observed for the control strains (Figure 1D). Note that an earlier study has indicated that the growth of the single Δ*hjc* mutant is not affected under these growth conditions (8).

### Hef forms an elongated dimer in solution

**Figure 2.**
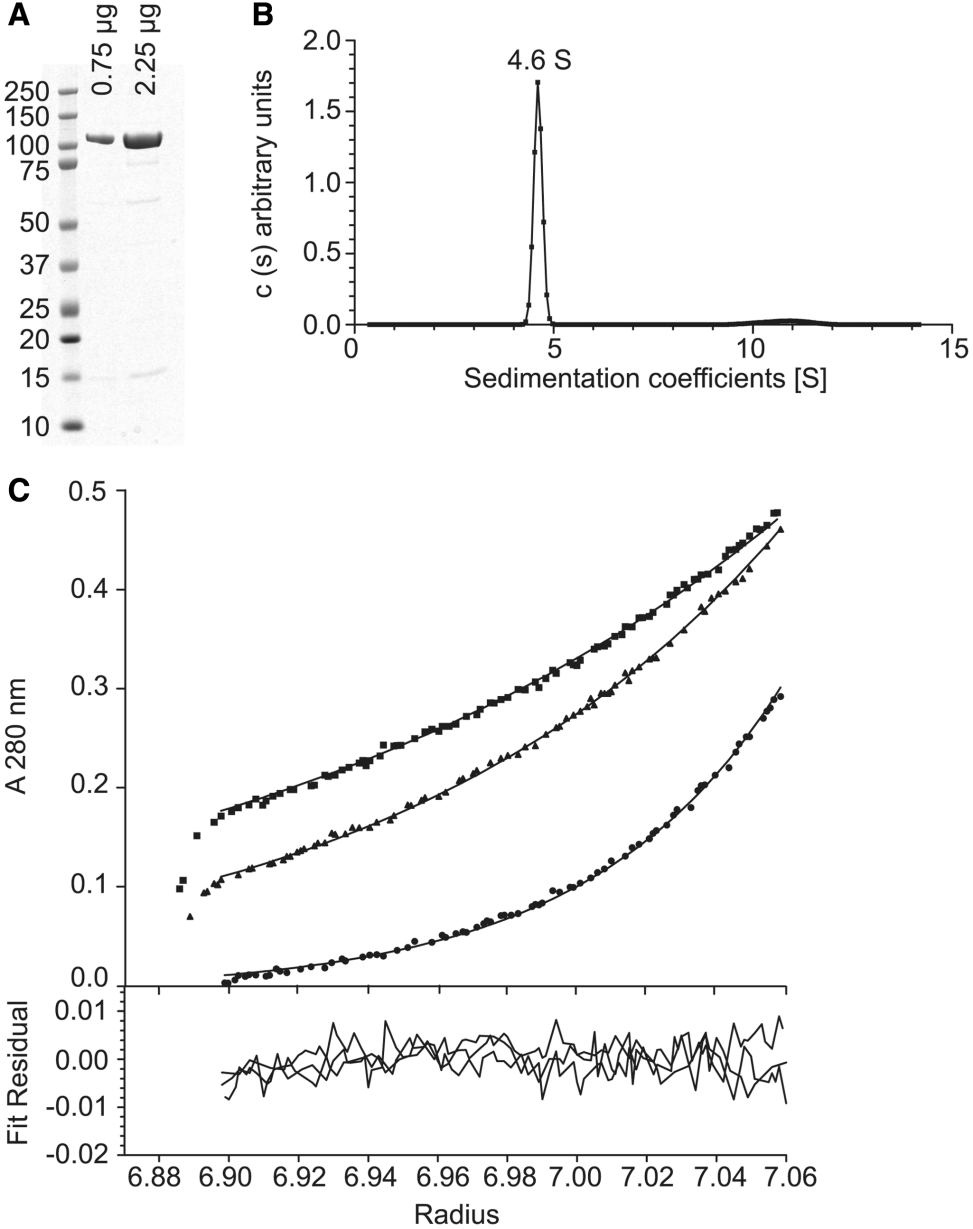
*In vitro* characterization of Hef oligomeric state and shape. (**A**) 0.75 and 2.25 µg of purified *H.volcanii* Hef holoprotein, separated on 4–12% SDS-PAGE. (**B**) Sedimentation velocity analysis of *Hvo*Hef at 20°C. The data recorded at 1 mg/ml in 2M KCl with 10 mM HEPES (pH 7.0) were fitted using SEDFIT software (22). (**C**) Sedimentation equilibrium analysis of *Hvo*Hef at 20°C. A *Hvo*Hef sample at 1 mg/ml in 2M KCl with 10 mM HEPES (pH 7.0) was used. Data recorded at 3564*g* (black square), 5132*g* (black triangle) and 10 649*g* (black circle) were fitted using species analysis model 1 of the SEDPHAT software.

To establish the oligomeric state and overall shape of the *Hvo*Hef protein, we expressed Hef with a six-histidine tag and purified it from its native host. For expression, a tryptophane-inducible promoter was used that was previously developed for this species (18). The Hef holoprotein was purified to ≈95% homogeneity using Ni-NTA agarose and gel filtration chromatography. Although a predicted monomeric molecular mass of *H. volcanii* Hef is 93.95 kDa, purified protein showed an apparent molecular weight of >100 kDa on SDS-PAGE (Figure 2A). This behavior is not unexpected as, owing to their reduced SDS-binding capacity, halophilic proteins tend to migrate slower than nonhalophilic marker proteins. To determine the apparent molecular weight and oligomeric state of the native Hef protein, AUC experiments were performed (Figure 2B and C). These sedimentation studies established a molecular weight of 184.5 ± 3.4 kDa, revealing that *Hvo*Hef is dimeric in solution with a sedimentation coefficient of 4.6S (S_20,W_ = 6.91S). The Hef-dimer has a frictional ratio (ƒ/ƒ^0^) of 1.76, indicative of a nonglobular elongated shape with a hydrodynamic radius of 6.65 nm (22).

### Hef–GFP forms fluorescence foci in living *H. volcanii* cells

Next, we studied the cellular localization of the functional GFP-tagged Hef protein in living *H. volcanii* cells. Cells expressing *hef*^+^*::gpf*^+^ were placed on a thin agarose slice, recovered with a glass coverslip and subjected to wide-field imaging with an Axio Observer ZEISS microscope with a 40×, 1.3 NA oil objective (0.26 µm resolution). Fluorescent foci were automatically detected within cells, and their fluorescence intensity was measured. All analysis steps were fully automated, allowing quantitative analyses of thousands of cells in the absence of a user bias and with extremely high statistical power.

**Figure 3.**
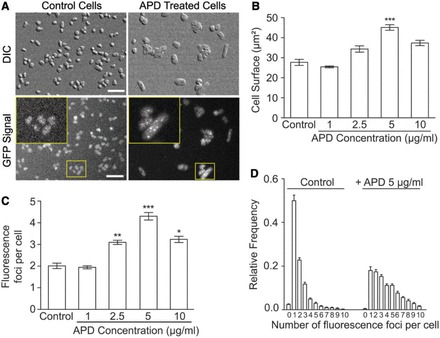
*In vivo* localization of GFP-labeled Hef in response to aphidicolin exposure. A total of 23 760 foci within 13 666 control cells and 15 299 foci within 3721 APD-treated cells were analyzed. (**A**) Pictures of DIC and GFP signal of *hef*^+^::*gfp*^+^ (HvRL37) cells under control conditions and after exposure to 5 µg/ml aphidicolin. Bar equals 10 µm. (**B**) Average cell surface of HvRL37 cells in response to increasing concentrations of aphidicolin. (**C**) Mean number of GFP-Hef labeled fluorescence foci in HvRL3) cells in response to increasing concentrations of aphidicolin. (**D**) Relative frequency of number of foci per individual cell. All error bars represent standard deviation (SD). n ≥ 3 experiments, *t*-test are performed in comparison to control without aphidicolin. ***Significantly different, *P* < 0.001; **Significantly different, *P* < 0.01; *Significantly different, *P* < 0.05.

As previously described (24), *H. volcanii* cells grown in rich media in the absence of drugs appeared pleiomorphic (Figure 3A), with an average cell surface of 28 ± 6 µm^2^ (n = 13 666). Although under these conditions autofluorescence signals were observed even in wild type cells not expressing GFP protein, analysis of 23 760 spots within 13 666 cells revealed specifically formed fluorescence foci only in cells containing Hef–GFP fusions. No foci were detected in wild type cells or in cells expressing nonfused GFP proteins (HvRL65 and HvRL66). Fifty percent (±11%) and 23% (±4%) of *hef*^+^::*gfp*^+^ cells had one or two foci, respectively, whereas only 2% (±1%) *hef*^+^::*gfp*^+^ cells did not form fluorescence foci at all. The average number of fluorescence foci measured under normal growth conditions, in the absence of any drugs, was 2.0 ± 0.5 foci (average of average foci number from 17 independent experiments). Using quantitative western immunoblot analyses with anti-GFP antibodies, we determined the GFP concentration in cells expressing nonfused monomeric or dimeric GFP and in *hef*^+^::*gfp*^+^ cells. We established that, under the conditions tested, our detection limit was ∼100 molecules per cell. The absence of detectable expression signals for the Hef::GFP fusion suggested that Hef::GFP is expressed at relatively low level (<100 molecules *per* cell) (Supplementary Figure S1C and D).

### Aphidicolin increases the cell size and the number of fluorescence foci

We then investigated the effect of the DNA synthesis inhibitor aphidicolin on the intracellular localization of the Hef::GFP fusions in *H. volcanii* cells. Exposure to increasing concentrations of aphidicolin increased the average cell size from 28 ± 6 µm^2^ (nontreated cells) to 45 ± 6 µm^2^ for cells exposed to 5 µg/ml of aphidicolin, thus indicating at least partial coupling between DNA replication and cell division in *H. volcanii* (Figure 3B). We observed that the fluorescence intensity per cell surface unit was about the same in cells exposed to aphidicolin and in control cells (Supplementary Figure S1B). In addition, the average number of fluorescence foci formed by GFP-tagged Hef proteins increased to a maximum of 4.3 ± 0.7 (n = 17) when exposed to 5 µg/ml aphidicolin (Figure 3C). Moreover, addition of 5 µg/ml of aphidicolin significantly changed the distribution pattern of the foci per individual cell compared with nontreated control cultures (Figure 3D).

### Effect of DNA damaging agents on Hef localization in living cells

**Figure 4.**
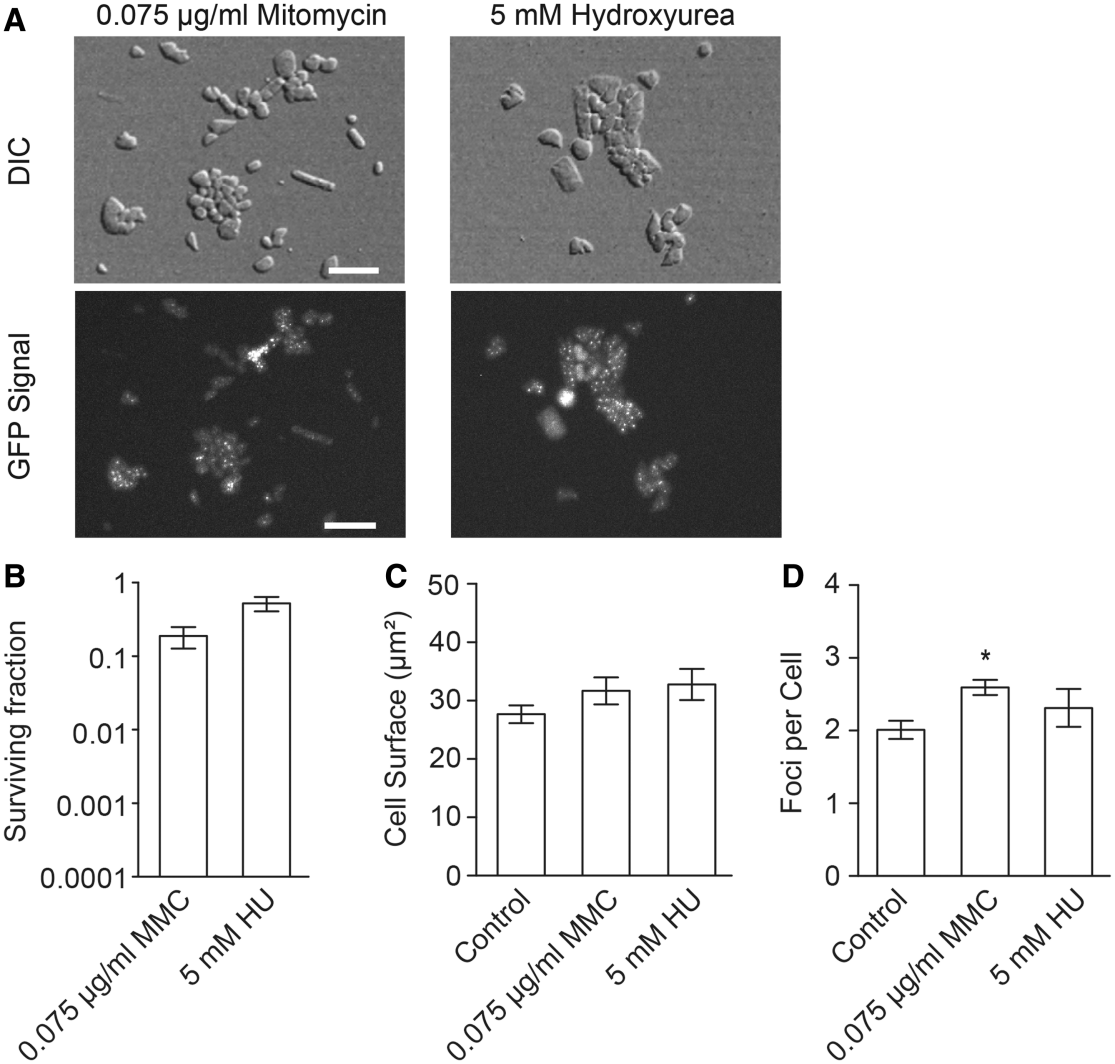
*In vivo* localization of GFP-labeled Hef in response to DNA damaging agents. A total of 3049 spots within 1492 HU-treated cells and 2738 spots within 1011 MMC-cells were analyzed. (**A**) Pictures of DIC and GFP signal of HvRL37 cells under control conditions and after exposure to 0.075 µg/ml MMC or 5 mM HU. Bar equals 10 µm. (**B**) Surviving fractions in response to exposure to drugs. (**C**) Average cell surface of HvRL37 in response to drug exposure. (**D**) Mean number of GFP-Hef labeled fluorescence foci in HvRL37 cells in response to exposure to drugs. All error bars represent SD. n ≥ 3 experiments, *t*-test are performed in comparison to control without aphidicolin. *Significantly different, *P* < 0.05.

To test whether the Hef::GFP localization observed in living cells was specific for aphidicolin, we also investigated the localization of Hef::GFP in cells treated with the cross-linking agent MMC (25), the double-strand break-causing agent phleomycin (26) and HU that decreases the size of the deoxyribonucleotide pool (27,28). Exposure to these agents was performed as described above for aphidicolin and several concentrations were tested to identify the compound concentration resulting in a similar level of cell death that was observed for 2.5 µg/ml aphidicolin. Concentrations of 0.075 µg/ml MMC and 5 mM HU did not result in marked changes in cell size (32 ± 4 µm^2^ and 35 ± 3 µm^2^, respectively) nor did they alter the average number of fluorescence foci per cell (2.6 ± 0.2 and 2.3 ± 0.6, respectively) compared with nontreated control cultures (3049 spots within 1492 HU-treated cells and 2738 spots within 1011 MMC-cells analyzed) (Figure 4). Treatment with 0.1 mg/ml phleomycin resulted into highly irregularly shaped cells that were not further analyzed in this study.

### Hef localization does not depend on Hjc

**Figure 5.**
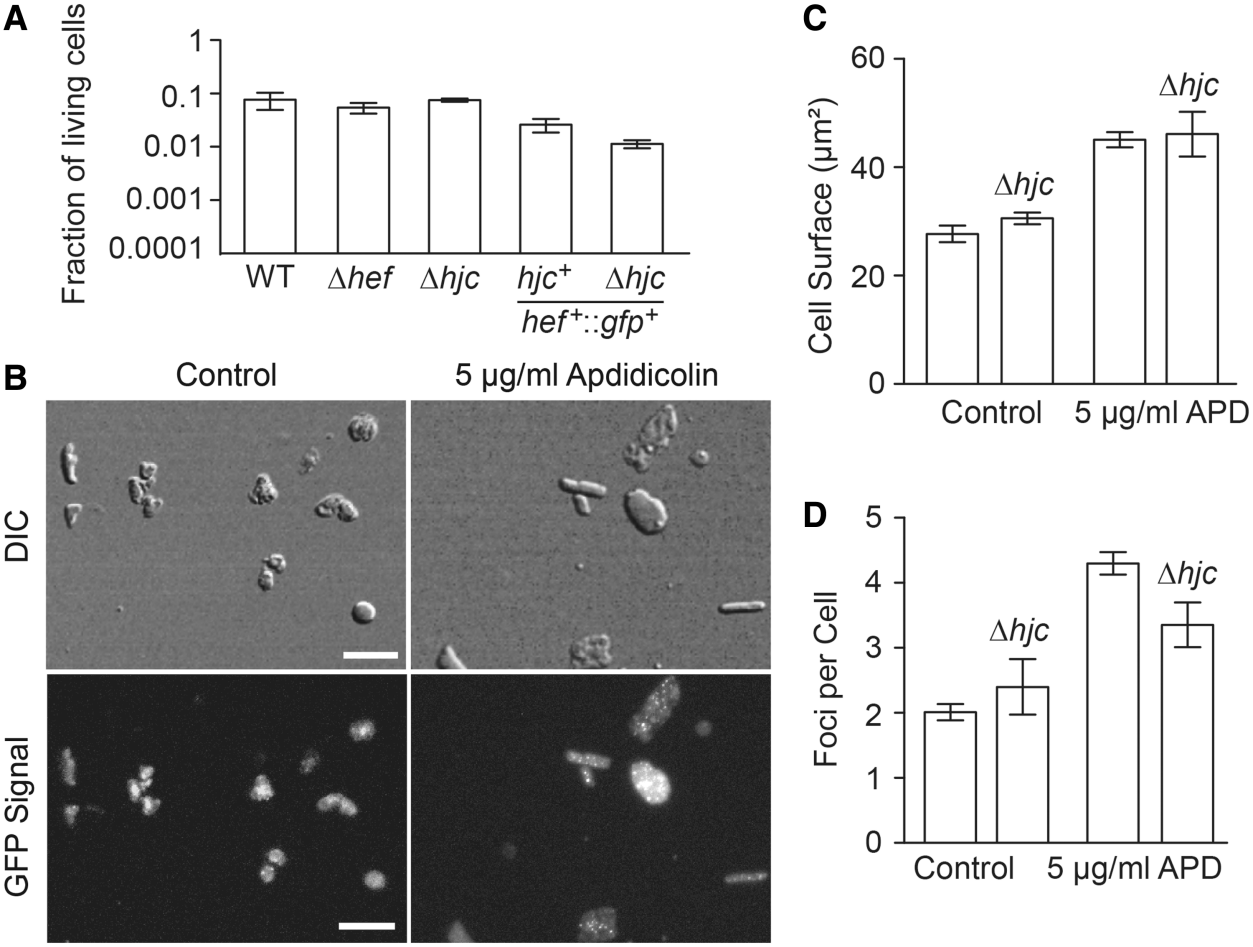
*In vivo* localization of GFP-labeled Hef in response to aphidicolin exposure in absence of Hjc. (**A**) Fraction of living cells in response to 5 µg/ml aphidicolin exposure. (**B**) DIC and GFP signal of *hef*^+^::*gfp*^+^Δ*hjc* (HvRL61) cells under control conditions and after exposure to 5 µg/ml aphidicolin. Bar equals 10 µm. (**C**) Average cell surface of *hef*^+^::*gfp*^+^Δ*hjc* cells in response to 5 µg/ml aphidicolin exposure. (**D**) Mean number of GFP-Hef labeled fluorescence foci in *hef*^+^::*gfp*^+^Δ*hjc* cells in response to 5 µg/ml aphidicolin exposure. All error bars represent SD. n ≥ 3 experiments.

Our previous genetic study indicated that Hef and Hjc cannot be simultaneously deleted, suggesting that they function in two parallel pathways (8). This synthetic lethality phenotype prompted us to investigate Hef::GFP localization in the absence of Hjc. Because growth of the *hef*^+^*::gfp*^+^Δ*hjc* strain is slow, we increased the drug exposure time to 70 h to expose this strain during seven to eight generations to 5 µg/ml aphidicolin. Our results indicated that the absence of Hjc did not influence cell viability, the cellular surface area or the mean number of fluorescence foci of the Hef:GFP fusion when compared with wild type and Δ*hef* cells (Figure 5). Thus, cellular function and localization of Hef is independent of the presence of Hjc.

### Two different populations of Hef::GFP fusion proteins are observed on replication inhibition

**Figure 6.**
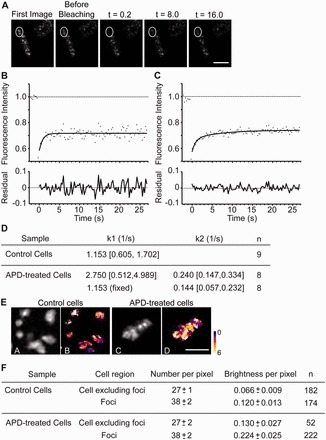
Dynamic localization of GFP-labeled Hef molecules at fluorescence foci. (**A**) Images of a representative cell in response to aphidicolin treatment for FRAP analysis. FRAP regions are shown by white circles. Time after photobleaching in seconds. Bar equals 5 µm. (**B**) Fluorescence recovery curve averaged for 9 control cells. Root-mean-square error (RMSE) = 0.0300 (**C**) Fluorescence recovery curve averaged for eight aphidicolin-treated cells. RMSE = 0.0155. (**D**) Diffusion constants (Confidence interval at 95%) calculated for GFP-labeled Hef diffusing molecules. (**E**) Images of representative cells for N&B analysis. Average intensity (A and C) and pseudo-coloured normalized brightness values (B and D) for representative control cells (A and B) and cells exposed to 5 µg/ml aphidicolin (C and D). Bar equals 5 µm. (**F**) Summary of results of N&B analysis ( ± SEM).

To reveal the intracellular dynamics of Hef localization, we performed FRAP experiments. For these studies, cells were immobilized on a glass cover slide coated with positively charged poly-D-lysine. Under these experimental conditions, wild type (H26) cells did not show any fluorescence signal. The averages of all individual measurements are shown in Figure 6. Cytoplasmic regions of *hef*^+^*::gfp*^+^ expressing cells were photobleached and measurements were performed individually for several cells. For cells grown in the absence of aphidicolin, a mono-exponential fit of recovery curve reflected the existence of one major population of diffusing Hef::GFP molecules, with a recovery constant estimated at 1.15 s^−^^1^ [with a 95% confidence interval (95% CI) from 0.60 to 1.70 s^−^^1^] (Figure 6A–D). Five seconds after photobleaching, maximally 35% of the fluorescence was recovered, with the remaining 65% corresponding to an immobile long-lived Hef::GFP fraction. As the surface of the bleached area is relatively large compared with the total cell surface (21% ± 5%), this potentially limits full fluorescence recovery on the timescale of this experiment. We also performed FRAP experiments on cells exposed to aphidicolin, including fluorescence foci in the photobleached regions. Five seconds after photobleaching, a maximum recovery of 44% of the bleached fluorescence signal is reached. In this case, a bi-exponential function was required for a robust fit of fluorescence recovery traces (Figure 6C). We expected one of the recovery phases to be similar to the one measured in nontreated cells. Indeed, we estimated using the bi-exponential fit a recovery constant of 2.75 s^−^^1^ (95% CI: 0.51–4.99), which is not statistically different from the diffusing population observed in control cells (see Figure 6D). In addition, a second, much slower phase with an apparent recovery constant of 0.24 s^−1^ (95% CI: 0.15–0.3) represented 34.7% [95% CI: 22.1–47.3] of the diffusing molecules. These results show that aphidicolin treatment slows down diffusion of Hef:GFP fusions in living *H. volcanii* cells.

### Clustering of Hef::GFP fusion proteins

To test whether a further decrease in diffusion of Hef::GFP molecules in the presence of aphidicolin could correspond to formation of aggregates and/or oligomerization of Hef, we performed N&B analysis. Fluctuation of fluorescence intensity in each pixel was measured, allowing the study of diffusion of fluorescent particles within one pixel. This method permits distinguishing the number of diffusing molecules and determining their molecular brightness reflecting their oligomeric state. Individual foci and the cell areas excluding foci were analyzed (Figure 6E).

Analyses performed on 182 control cells with 174 fluorescent foci, as well as on 52 aphidicolin-treated cells with 222 foci, showed that the number of diffusing molecules (outside of foci) per pixel is similar in aphidicolin-treated and control cells (27 ±1 and 27 ± 2 diffusing molecules, respectively) (Figure 6F). In the fluorescence foci, the average numbers of detected GFP-tagged Hef proteins per pixel increased ∼40% both in nontreated and treated cells (38 ± 2 and 38 ± 2 molecules, respectively). Strikingly, these analyses also revealed a substantially higher brightness of the Hef::GFP molecules at the fluorescence foci in comparison with the rest of the cell, indicating clustering/co-localization and/or oligomerization of individual Hef protein complexes at foci (Figure 6F).

## DISCUSSION

In this study, we have investigated the *in vivo* role of Hef in the euryarchaeon *H*. *volcanii*. Hef is the unique archaeal member of the XPF/MUS81/FANCM family of structure-specific endonucleases and is essential for cell viability in the absence of the Holliday junction resolvase Hjc or the recombinase RadA, indicating that Hef and Hjc/RadA provide alternative means to restart arrested DNA replication forks (8). We have used advanced microscopy techniques to investigate the cellular dynamics of a GFP-labeled Hef protein expressed at physiological level from the chromosomal locus in living *H*. *volcanii* cells. Our results indicate that the GFP-tagged Hef protein is functional in repair of DNA damages caused by MMC or aphidicolin (Figure 1). We nevertheless note that Hef may have additional functions in DNA repair and/or replication in *H. volcanii*, as in the absence of Hjc, this GFP-fusion protein results in a hypomorphic phenotype (Figure 1).

Combining wide-field imaging and quantitative image analysis, we have shown that GFP-labeled Hef proteins formed fluorescent localization foci under normal growth conditions. The number of these fluorescence foci was substantially increased by aphidicolin that blocks the elongation state of DNA replication in halophilic archaea, including *H*. *volcanii* (23) (Figure 3). We verified that the formation of fluorescence foci by GFP-labeled Hef proteins was not simply the consequence of DNA damage. For instance, the number of Hef localization foci was not increased by MMC or HU treatments. Also, while in cells exposed to phleomycine, a drastic effect on cell survival and morphology was seen, no GFP-labeled Hef fluorescence foci could be observed (Figure 4). The fluorescence foci that we observed in the absence and presence of aphidicolin indicate that Hef specifically localizes at arrested replication forks. An average of 2.0 ± 0.5 GFP-labeled Hef foci was observed in control cells and (in average) 4.3 ± 0.7 upon exposure to 5 µg/ml aphidicolin (up to 10 foci/cell have been infrequently observed). Such a high number of arrested replication forks per cell is feasible as the circular chromosome of *H. volcanii* carries multiple replication origins (29) and is highly polyploid (30). Our results are also consistent with a coupling of DNA replication and cell division in *H. volcanii*, as the cell surface significantly increased upon inhibition of DNA replication.

To further study *in vivo* the response of *H. volcanii* to inhibition of DNA replication, we have performed the first FRAP experiments in any archaeal cell to date. In the absence of aphidicolin, one major population of Hef::GFP molecules was observed with an apparent diffusion rate estimated at 0.8–2.3 µm^2^/s (Figure 6). This diffusion coefficient is dramatically lower than the one measured for free GFP in the cytosol of *E. coli* DH5α (7.7 ± 2.5 µm^2^/s) (31). An eight-fold increase in protein size is expected to lower the particle diffusion constant by a factor of two (32). Assuming similar physicochemical constraints for protein diffusion (e.g. viscosity) of bacterial and archaeal cytosols, we expected that the *Hvo*Hef::GFP dimer should have an approximate diffusion coefficient of 3–4 µm^2^/s. The measured diffusion coefficient is significantly lower than the predicted value, indicating that the peculiar elongated shape revealed by AUC analyses (Figure 2), considerable physicochemical constraints and/or transient interactions with cellular components, in particular with DNA, drastically limit protein diffusion of Hef in the cytosol of *H. volcanii*.

In the case of aphidicolin-treated cells, a second, much slower population with an apparent diffusion coefficient of 0.08–0.31 µm^2^/s was also detected that represents 30–40% of the total fluorescent molecules (Figure 6C). This population may correspond to slowly diffusing molecules brought about by aphidicolin treatment, or may represent an altered association and/or dissociation of Hef proteins from arrested or collapsed replication forks induced by aphidicolin. We favor the latter possibility, as N&B analyses indicated that the average number of GFP-tagged Hef proteins per pixel is increased by ∼40% in fluorescence foci compared with diffusing molecules. This indicates that the GFP fusions at the foci either have a higher oligomeric state or that several molecules are co-diffusing. These two nonexclusive hypotheses can explain the presence of apparently slowly diffusing, but brighter fluorescence, foci formed by Hef::GFP that are likely to interact with DNA. These observations also provide experimental evidence that Hef is actively recruited at arrested and/or collapsed replication forks. This is similar to recent observations that FANCM proteins protect stalled replication forks in human cells (33). Note that in *Pyrococcus* species, Hef is known to interact with Proliferating cellular nuclear antigen, the nexus of DNA replication (11) and that this interaction might be evolutionarily conserved between hyperthermophilic and halophilic euryarchaea. Consequently, the identification of interaction partners for Hef proteins could constitute a highly relevant starting point for understanding the molecular details of Hef recruitment at arrested replication forks.

**Figure 7.**
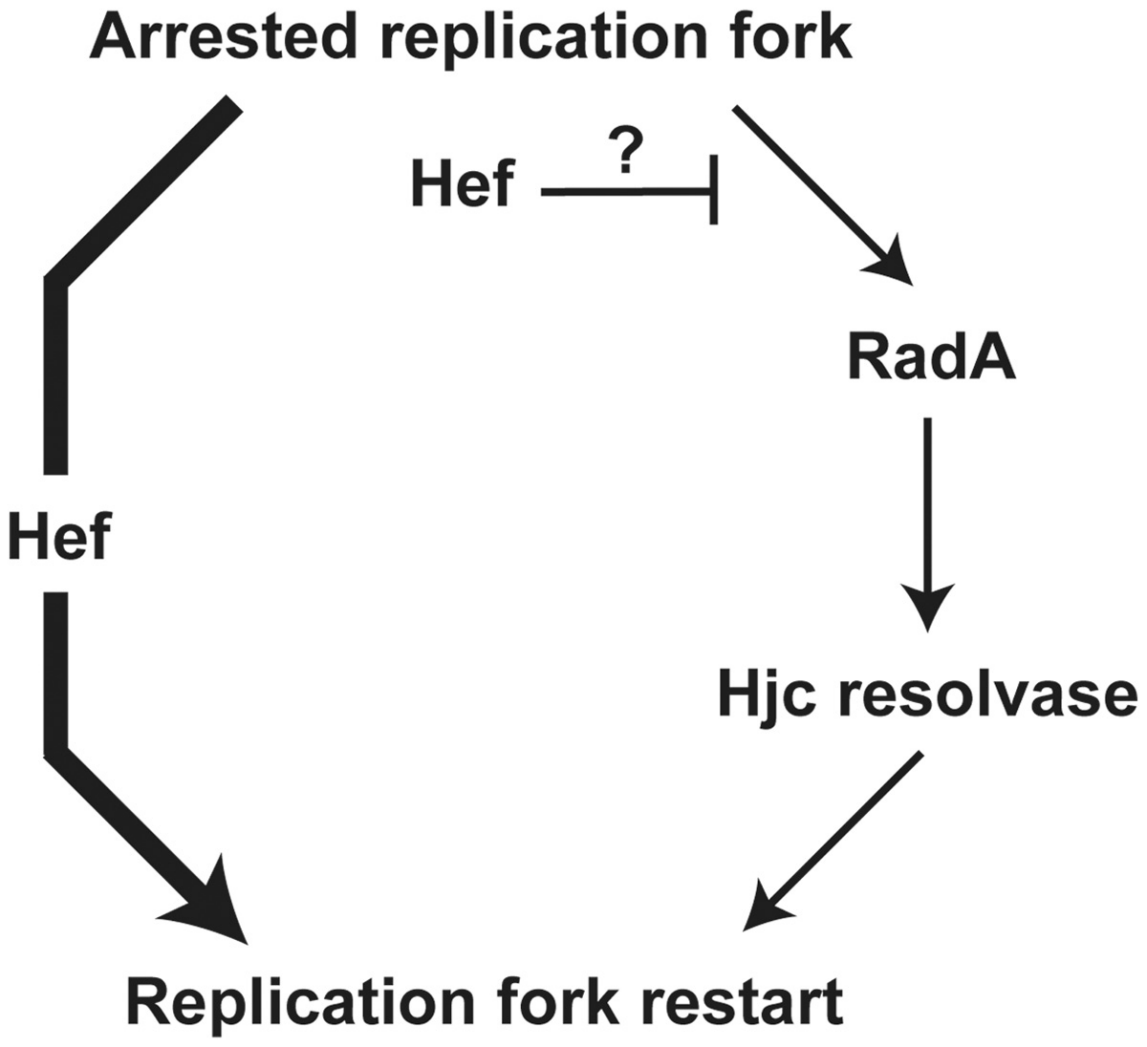
Model for replication restart in *H.volcanii*. Two alternative pathways allow replication restart: one is dependent on the homologous recombination proteins Hjc and RadA (pathway on the right) and one is independent of homologous recombination (pathway on the left). Our data show that Hef has a dominant role during replication restart, even in the presence of Hjc, and are also compatible with Hef, preventing the access of recombination proteins at arrested forks.

Even though Hef and the recombination proteins Hjc/RadA provide alternative ways for replication restart in *H. volcanii* (8), the absence of Hjc did not influence the number of fluorescence foci formed by Hef::GFP, neither in control nor in aphidicolin-treated cells (Figure 5). This indicates that in living cells, Hjc does not bind, in appreciable quantity, the same substrates as Hef and raises the possibility that Hef has a dominant role during replication restart, even in the presence of Hjc (Figure 7). Hef may also prevent the access of recombination proteins at arrested forks. This scenario implies the existence of a replication restart pathway that is independent of homologous recombination, which is supported by the viability of a Δ*radA* mutant deficient for homologous recombination. Recently, it has been shown that FANCM proteins, eukaryotic homologs of Hef, can prevent homologous recombination (13,34–37). The role of Hef in possibly controlling a homologous recombination-dependent replication restart pathway by processing arrested replication forks remains yet to be elucidated.

In conclusion, our results revealed that Hef proteins form specific localization foci in a highly dynamic manner in response to halting DNA replication. Our *in vivo* imaging studies have established *H. volcanii* as a unique model system for archaeal cellular biology, in particular, for investigating replisome dynamics and genome stability in living cells.

## SUPPLEMENTARY DATA

Supplementary Data are available at NAR Online.

## FUNDING

Funding for open access charge: ANR, CNRS.

*Conflict of interest statement*. None declared.

## Supplementary Material

Supplementary Data
