# Supplementary material for: Intracellular dynamics of archaeal FANCM homologue Hef in response to halted DNA replication
Source: Nucleic Acids Res. 2013 Sep 17;41(22):10358–70. doi: 10.1093/nar/gkt816 (PMC3905845; doi:10.1093/nar/gkt816)
Supplement: Supplementary Data [file supp_41_22_10358__index.html]

Intracellular dynamics of archaeal FANCM homologue Hef in response to halted DNA replication — Intracellular dynamics of archaeal FANCM homologue Hef in response to halted DNA replication — Supplementary Data 

# Intracellular dynamics of archaeal FANCM homologue Hef in response to halted DNA replication

## Supplementary Data

files

**Files in this Data Supplement:**

- Supplementary Data - pdf file
