# Supplementary material for: Intracellular dynamics of archaeal FANCM homologue Hef in response to halted DNA replication
Source: Nucleic Acids Res. 2013 Sep 17;41(22):10358–70. doi: 10.1093/nar/gkt816 (PMC3905845; doi:10.1093/nar/gkt816)
Supplement: Supplementary Data [file supp_gkt816_nar-01925-d-2013-File009.pdf]

## SUPPLEMENTARY FIGURE

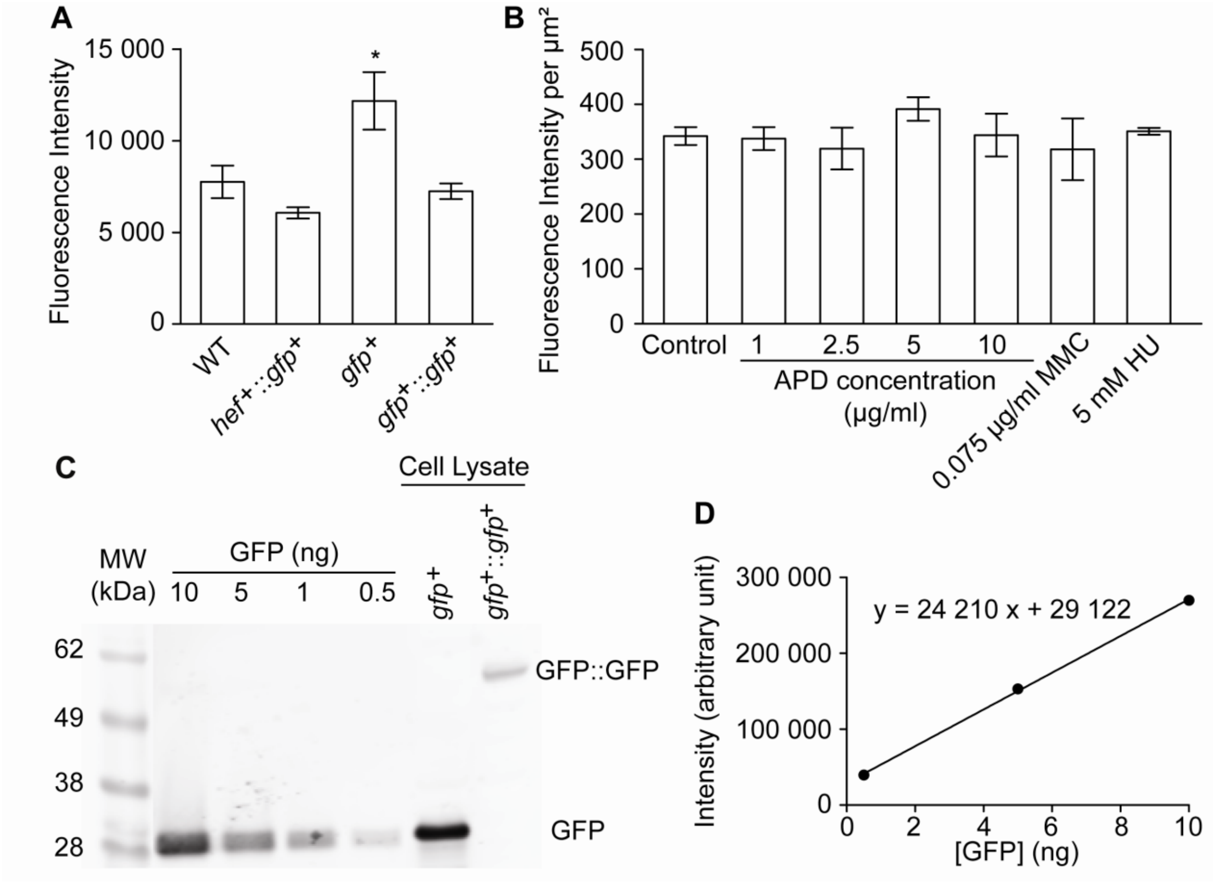


**Figure S1: Quantification of Hef::GFP expression.** (A) Histograms representing the average fluorescence intensity per cell measured in WT (H26), *hef*$^+$::*gfp*$^+$ (HvRL37), *gfp*$^+$ (HvRL65) and *gfp*$^+$::*gfp*$^+$ (HvRL66) cells. Errors bars represent SD. n experiments ≥ 3. T test to compare values measured in GFP-expressing cells to the value measured in WT cells. * means significantly different, $p<0.05$. (B) Histograms representing the fluorescence signal per cell surface unit measured in *hef*$^+$::*gfp*$^+$ (HvRL37). Errors bars represent SD. n experiments ≥ 3. (C) Western immunoblot using anti-GFP antibody. To quantify the amount of GFP in the lysates of *gfp*$^+$ (HvRL65) and *gfp*$^+$::*gfp*$^+$ cells (HvRL66) a calibration curve we established (shown in D) using known quantities of purified rGFPuv (Clontech).
